# The Affective Neuroscience Personality Scales: Linking the adjective and statement-based inventories with the Big Five Inventory in English and German-speaking samples

**DOI:** 10.1017/pen.2021.6

**Published:** 2022-03-23

**Authors:** Dmitri Rozgonjuk, Kenneth L. Davis, Cornelia Sindermann, Christian Montag

**Affiliations:** 1 Department of Molecular Psychology, Ulm University, Ulm, Germany; 2 Institute of Mathematics and Statistics, University of Tartu, Tartu, Estonia; 3 Pegasus International, Greensboro, NC, USA

**Keywords:** Affective Neuroscience Personality Scales, Big Five, Personality traits, ANPS-AR

## Abstract

Jaak Panksepp’s Affective Neuroscience Theory is of high relevance not only for a better understanding of affective brain disorders but also in personality research. To make Panksepp’s theory more accessible for psychologists and psychiatrists, Davis, Panksepp, and Normansell (2003) developed the Affective Neuroscience Personality Scales (ANPS). These scales assess the manifestation of the primary emotional traits in humans based on a personality trait approach. Given their putative foundation in old subcortical areas in the brain, these primary emotional traits (assessed via the ANPS) could represent the evolutionarily oldest manifestations of personality (but this notion is still a matter of a debate). However, the ANPS inventories were based on using contextual items (e.g., about specific attitudes, behaviors, and feelings in specific situations). Recently, an adjective-based ANPS (ANPS-Adjective Ratings or ANPS-AR) was developed for a less context-dependent and more efficient assessment of Panksepp’s primary emotional systems in humans for use by both individuals and independent observer raters. The present work introduces the first German version of the ANPS-AR. Moreover, the current work investigates the original and ANPS-AR versions of the ANPS and their associations with the Big Five personality traits in two independent English- and German-speaking samples. The results show that the ANPS measures are very similarly correlated with the Big Five personality traits across different samples and scales. This work replicates the previous findings in an English version, and demonstrates the reliability and validity of the adjective-based German ANPS-AR.

Jaak Panksepp’s Affective Neuroscience Theory is a prominent emotion theory (Davis & Montag, 2019; Panksepp, 1998, 2011). In short, Panksepp observed seven primary emotional systems not only in studies applying electrical brain stimulation but also in lesion studies and pharmacological challenges. These seven primary emotional systems are divided into positive and negative emotional systems, and are called SEEKING, LUST, CARE, PLAY (positive emotions), and RAGE (also operationalized as ANGER), FEAR, PANIC/GRIEF (also operationalized as SADNESS; negative emotions). The positive emotions are prototypically experienced as pleasant affects that signal survival. The negative emotions are typically experienced as unpleasant affects and, correspondingly, signal survival risks. Please note that such a categorization into positive and negative affect has been criticized by some researchers as too simplistic because, for instance, the experience of LUST might also elicit negative affect in some individuals, perhaps depending on the cultural context. For example, see the work by Cowen and Keltner (2017) who have outlined some problems regarding the complexities of categorizing human emotional experience.

Panksepp’s emotion theory is not only of high interest in basic emotion research, but is also relevant in clinical work (Panksepp, 2006). Imbalances in and between primary emotional systems can contribute to the development and maintenance of psychopathology: for instance, ANPS measures showing low SEEKING and high SADNESS have been linked to depressive tendencies (Montag, Widenhorn-Müller, Panksepp, & Kiefer, 2017; Panksepp & Watt, 2011). In addition to emotion research and clinical studies, Panksepp’s theory has been utilized in personality science. For instance, Davis et al. (2003) and Marengo, Davis, Gradwohl, and Montag (2021) have demonstrated consistent associations between ANPS and Big Five personality measures. Also, Montag and Panksepp (2017a, 2017b) hypothesized that the subcortically based primary emotional systems might represent the phylogenetically oldest foundation of the lexically derived Big Five personality traits.

## 1.1 Assessing primary emotional systems with self-reports

Self-reports are very useful in studying human emotions, because other methods (e.g., neurobiological) are more expensive in terms of time and resources and often pose ethical limitations. Also, self-reports are holistic and consider how individuals see themselves. Therefore, Jaak Panksepp was interested in developing the Affective Neuroscience Personality Scales (ANPS) for use in clinical research. For the most recent version (ANPS 3.1) and examples of clinical studies using the ANPS see the work by Montag et al. (2021).

Short versions of the ANPS have been developed, too; for example, BANPS (33 items; Barrett, Robins, & Janata, 2013) and ANPS-S (36 items; Pingault, Falissard, Côté, & Berthoz, 2012) which were primarily shortened inventories derived from the original ANPS (for a recent comprehensive review, see Montag et al., 2021). The ANPS was based on a background of neurobiological studies mentioned above, and not derived via a lexical approach. Aiming to facilitate observer ratings and efficiency in general, Montag and Davis (2018) published a short adjective-based ANPS-AR, an ANPS version with 24 items. And, given its reliance on adjective-based descriptions, it could potentially reflect given emotions more directly and intuitively. However, the English-language version of ANPS-AR is relatively new and, therefore, there is a lack in validation studies demonstrating the similarity with its longer counterpart.

## 1.2. Present study

The main aim of the current study is to examine the psychometric properties of the ANPS-AR and its links to a Big Five personality trait measure. Of importance, ANPS measures have been correlated with the Big Five personality traits in several earlier works. A recent meta-analysis by Marengo et al. (2021) among others provides a summary of these links (the meta-analysis does not include studies using the ANPS-AR, as these were not available until after the publication of that paper). For instance, high SEEKING has been associated with higher Openness to Experience, higher PLAY with higher Extraversion, higher CARE/lower ANGER was linked to higher Agreeableness, and higher ANGER/FEAR/SADNESS was associated with higher Neuroticism.

Therefore, the aims of the current report were to build on the results reported in Montag and Davis (2018) to investigate the relationships between the long ANPS and short ANPS-AR measures and the Big Five personality traits, and to validate the German version of the ANPS-AR by doing so. To meet these aims, data from two studies are used. Study 1 is an English-language based survey which included the ANPS-AR and the BFI-44 (see details of the scales in Section 2.2). In Study 2, German-speaking participants filled out the original version of the ANPS (Davis et al., 2003), the ANPS-AR, and the 44-item Big Five Inventory (BFI; see details in Section 2.2). Because some of the subscales of the ANPS-AR have shown rather low internal consistencies in previous work (Montag & Davis, 2018), four additional items were included for the ANPS-AR in the German language Study 2. The correlations in these two studies with slightly different measures are presented to demonstrate the links with the Big Five personality traits. We expected that the correlations between the Big Five domains and six scales of the ANPS would be rather similar across these studies, which used slightly different ANPS-AR variants.

## 2. Method

### 2.1 Samples and procedure

In the current work, we used the data from two different studies. Study 1 was conducted in English language, while Study 2 was in German language. Both datasets are parts of larger projects where personality measures were included. Both projects encompassed online surveys where participation was anonymous and voluntary, and incentivized by providing feedback on participants’ responses (e.g., on personality traits in comparison to other participants). Both projects were approved by the Institutional Review Board of Ulm University, Germany.

In Study 1, the sample was recruited via print, social, and radio media. The larger project focused on individual differences in social media use. Of importance to the current work, Study 1 included basic socio-demographic data (e.g., age and gender), as well as responses to the BFI-44 and ANPS-AR in English language. In total, 497 people responded to the questionnaires. However, in the current work, we focused on adults. Therefore, 88 people who were under 18 or had implausible age values (e.g., 441) were excluded from the analyses. Next, we excluded data of participants who had indications of careless response patterns, for example, answering most of the questions of a given personality inventory with the same response. Participants who had less than 20 consecutive responses on the BFI-44 and less than 12 consecutive responses on the ANPS-AR measure were included. The effective sample comprised 405 adults (age M = 26.42; SD = 7.67; 300 men, 105 women).

The aim of the larger project of Study 2 was to investigate smartphone and social media use. As with Study 1, the online platform was promoted via different media channels, among other social media, but also TV and radio. The initial sample comprised N = 1951 participants. The general data cleaning procedure followed similar steps as in Study 1: we first included only participants who were at least 18 years old, had plausible age values, and did not have missing data in variables of focus in this work. Subsequently, we checked for careless response patterns in personality questionnaires. Participants who had less than 20 consecutive responses on the BFI-44, less than 12 consecutive identical responses on the ANPS-AR measure, and less than 40 identical consecutive responses on the 110-item ANPS scale were included. After this, we sampled 500 people (250 men and 250 women) for a gender-balanced sample to form the effective sample (age M = 25.97, SD = 7.79). Relevant to this work, Study 2 included socio-demographic questions, the BFI-44, the original version of the ANPS, and the ANPS-AR (including four additional items and labeled the ANPS-AR-28).

### 2.2 Measures

#### 2.2.1 The Big Five personality traits

To assess Big Five personality traits, both studies used the BFI-44. The English-language original BFI was developed by John, Donahue, and Kentle (1991), and it initially included 45 items with a response scale of 1 = “disagree strongly” to 5 = “agree strongly.” The BFI was adapted to German by Rammstedt and Danner (2017) and also applies a five-point response scale (1 = “very inapplicable” to 5 = “very applicable”). The BFI consists of five domains (number of items is presented in parentheses): Openness to Experience (10); Conscientiousness (9); Extraversion (8); Agreeableness (9); and Neuroticism (8). As suggested in Rammstedt and Danner (2017), the 45th item added in the German version is not typically used to compute scale scores. Moreover, to keep the scores comparable across English- and German-language studies, we also excluded the 45th item from the German-speaking sample. Reverse-coded items were first recoded, and average scores for the BFI-44 domains were computed. The descriptive and internal consistency statistics for domains are presented in Table 1.

**Table 1.** Descriptive statistics for the original ANPS, ANPS-AR, and the Big Five measures

Personality scale	Study 1 (N = 405)			Study 2 (N = 500)			Mean comparison			
BFI-44	M	SD	α	M	SD	α	*t*	*df*	*p*	*d*
Openness	3.69	0.62	.76	3.55	0.70	.81	3.074	896.53	.002	.203
Conscientiousness	3.23	0.71	.80	3.38	0.67	.83	−3.238	842.92	.001	.218
Extraversion	2.86	0.83	.84	3.22	0.81	.87	−6.596	855.55	<.001	.442
Agreeableness	3.62	0.65	.75	3.52	0.59	.72	2.425	826.06	.016	.164
Neuroticism	2.93	0.89	.86	2.97	0.80	.85	−0.769	818.85	.442	.052
**ANPS**										
seeking^a^	5.21	0.93	.56	5.36	0.85	.50	−2.386	830.67	.017	.161
seeking^b^	–	–	–	5.18	0.89	.74	–	–	–	–
SEEKING	–	–	–	2.85	0.38	.74	–	–	–	–
fear^ab^	3.94	1.50	.85	4.03	1.11	.72	−.959	727.45	.338	.066
FEAR	–	–	–	2.72	0.54	.84	–	–	–	–
care^a^	5.08	1.04	.69	5.44	1.10	.72	−5.033	880.83	<.001	.335
care^b^	–	–	–	5.44	1.03	.88	–	–	–	–
CARE	–	–	–	2.85	0.47	.75	–	–	–	–
anger^ab^	3.58	1.11	.55	2.95	1.24	.76	8.072	893.22	<.001	.534
ANGER	–	–	–	2.47	0.51	.77	–	–	–	–
play^ab^	5.32	1.02	.72	5.55	0.95	.83	−3.417	837.20	<.001	.230
PLAY	–	–	–	2.95	0.42	.73	–	–	–	–
sadness^ab^	4.13	1.38	.75	4.14	1.29	.79	−.167	839.31	.868	.011
SADNESS	–	–	–	2.52	0.45	.75	–	–	–	–

Uppercase ANPS domain names reflect the values for the original ANPS scale, while lower-case ANPS domain names reflect the values for the ANPS-AR variants.

a The domain score is for the ANPS-AR (English).

b The domain score is for the ANPS-AR-28 (German).

ab The domain is included in both ANPS-AR and ANPS-28. Theoretical range of the scales is min(ANPS-AR/ANPS-AR-28/ANPS) = 1, max(ANPS-AR/ANPS-AR-28) = 7, and max(ANPS) = 4.

#### 2.2.2 Primary emotional traits

To assess the primary emotional systems from a trait perspective (e.g., primary emotional traits), we used both the original ANPS and the adjective-ratings-based ANPS-AR.

The original 110-item ANPS used in this study assesses six primary emotional systems, in line with the Affective Neuroscience Theory (Panksepp, 1998, 2011). In this scale, the primary emotional systems can be divided into positive emotions (SEEKING, PLAY, CARE) and negative emotions (ANGER, FEAR, SADNESS). Each scale was assessed by 14 items responded to on a four-point scale (1 = “strongly disagree” to 4 = “strongly agree”). Of note, the original ANPS includes additional subscales that were not used in the current study, and the original ANPS does not include a LUST scale.

The ANPS – Adjective Ratings (ANPS-AR; Montag & Davis, 2018) assess the six primary emotional traits, assessed in the ANPS (SEEKING, ANGER, FEAR, CARE, SADNESS, PLAY) on a seven-point response scale (1 = “very inaccurate” to 7 = “very accurate”).

In Study 1, we used the English version of the ANPS-AR (Montag & Davis, 2018) with 24 items. The detailed procedure for scale development can be found in Montag and Davis (2018). To shortly summarize, the adjectives were drawn from a pool of Big Five adjectives that had the highest correlations with the ANPS. The instruction for the participants was as follows: “Please indicate how accurately the following words describe you.”

In Study 2, the ANPS and the ANPS-AR were presented in German language (translated following the standard back-translation procedure), with the ANPS-AR including two additional items for both the SEEKING and CARE subscales compared to the English version. Among others, these items were added due to psychometric reasons, as previous work has shown that, for instance, the SEEKING subscale had lower internal consistency (Montag & Davis, 2018), and preliminary German data suggested also that this would increase the reliability. Please note that in the German version we also used a seven-point response scale, but with a slightly different wording (1= “very inaccurate” to 7 = “very accurate”). The instruction here was as follows: “In general, I am …”. We also want to note that this wording may not be ideal, because one of the items in ANPS-AR, namely “jokes around,” does not sound grammatically correct. Therefore, we propose a better wording in the questionnaires file enclosed in Supplementary Materials.

The scale scores in all inventories were averages of given scale items. The descriptive and internal consistency statistics for these variables for the effective samples are presented in Table 1.

### 2.3 Analysis

The data were analyzed in R software v 4.1.1 (R Core Team, 2021). We firstly computed descriptive statistics (mean, standard deviation) for the averaged scale scores. Then, we computed the internal consistency statistics (Cronbach’s alphas) for each domain score with the *psych* package v 2.1.9 (Revelle, 2021). Finally, Pearson correlation coefficients with 95% confidence intervals between the Big Five and ANPS domain scores were computed with the *psych* package v 2.1.9 (Revelle, 2021). The confidence intervals were found based upon the sample sizes using the conventional *r*-to-*z* Fisher transformation and the normal distribution (Revelle, 2021).

## 3. Results

### 3.1 Descriptive statistics

The descriptive statistics for scales are displayed in Table 1. The descriptive statistics in Table 1 show that the two samples differ on several personality traits. The German-language sample scores higher in Conscientiousness and Extraversion, and lower in Openness and Agreeableness than the English-language sample. With regard to ANPS-AR, the German-language sample scores higher than the English-language sample in SEEKING, CARE, and PLAY, and lower on ANGER.

It can also be observed that SEEKING domain from the ANPS-AR with 24 items in both language versions (as well as ANGER in ANPS-AR English version) has low internal consistencies. However, including two additional items to the SEEKING scale (ANPS-AR-28) improves the internal consistency. Furthermore, the internal consistencies of the original 110-item ANPS and the ANPS-AR (especially after including additional items) domains are mostly of rather similar magnitude.

### 3.2 Correlations between the ANPS and the Big Five measures

**Figure 1.**
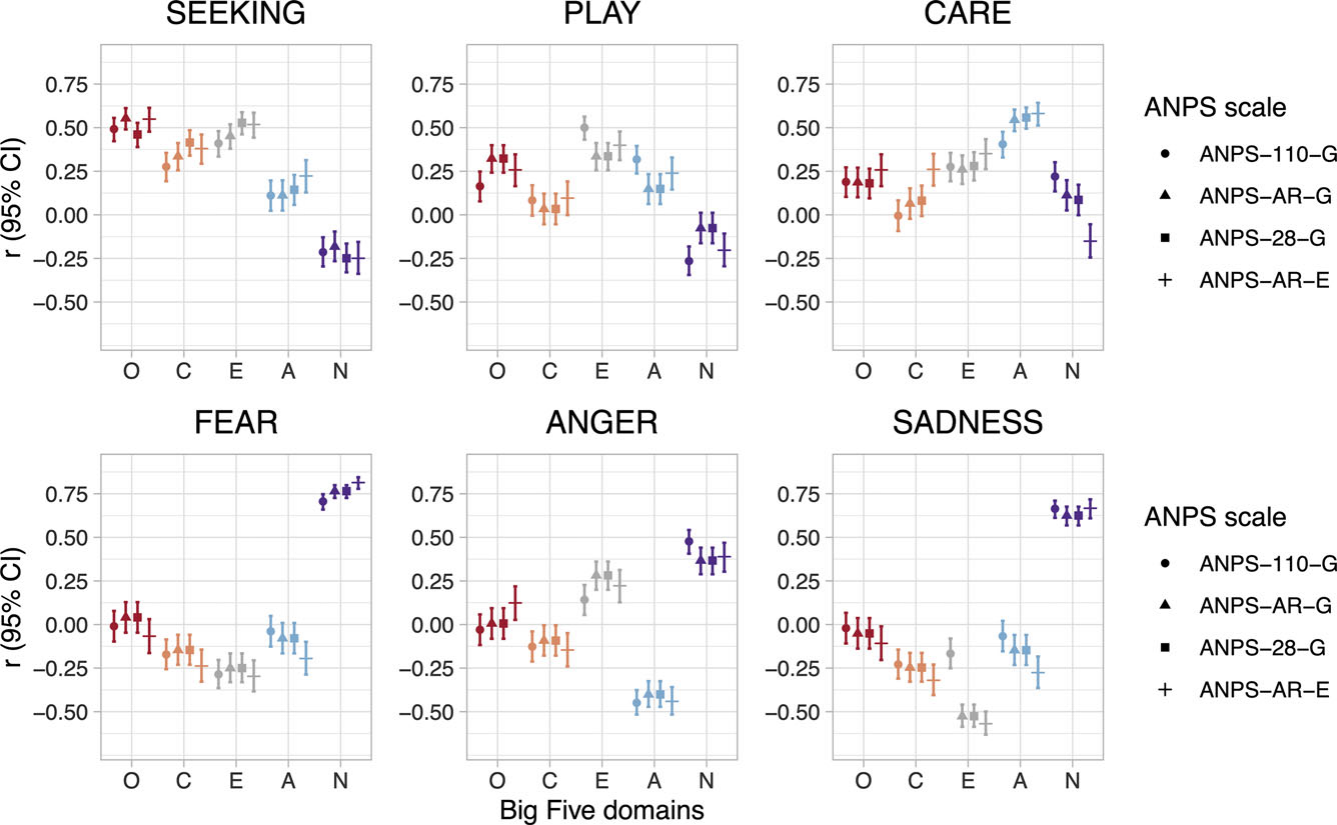
Pearson correlation coefficients (with 95% CIs) between the Big Five domains and ANPS measures. O: Openness to Experience; C: Conscientiousness; E: Extraversion; A: Agreeableness; N: Neuroticism; ANPS-110-G: German ANPS-110; ANPS-AR-G: German ANPS-AR; ANPS-AR-E: English ANPS-AR; ANPS-28-G: German ANPS-AR with two additional items for both the SEEKING and CARE scales.

The correlation analysis results are displayed in Figure 1. The numeric depiction of Pearson correlation coefficients is included in the Supplementary Materials.

The results in Figure 1 demonstrate that, in general, the correlations between the Big Five and the ANPS domains show similar patterns across samples and measures. Yet, even though the correlations and the 95% confidence intervals tend to overlap when different measures are compared, there seems to be a larger difference in Extraversion correlations between the German original ANPS scales and the German ANPS-AR. Especially, the ANPS-AR SADNESS scale has a much stronger negative correlation (*r* = −.526) with Extraversion than the original ANPS SADNESS scale has with Extraversion (*r* = −.167). There are also differences between how the ANPS-AR and ANPS PLAY scales correlated with Openness to Experience, Agreeableness, and Neuroticism.

With regards to the associations between the Big Five and ANPS domains, some patterns seem to be robust across different samples and measures. Openness to Experience is generally positively correlated with SEEKING, PLAY, and CARE. Conscientiousness is positively correlated with SEEKING, and negatively with FEAR, ANGER, and SADNESS. Extraversion is positively linked to SEEKING, PLAY, CARE, and ANGER, and negatively with FEAR and SADNESS. Agreeableness correlates positively with SEEKING, PLAY, and CARE, and negatively with ANGER. Finally, Neuroticism is negatively associated with SEEKING and PLAY, and positively correlated with FEAR, ANGER, and SADNESS.

## 4. Discussion

The aims of the current work were to investigate the associations between traits measured by different ANPS variants and the Big Five personality traits, and to validate the German version of ANPS-AR by doing so. To meet these aims, we used data from two different studies conducted in different languages. We expected that regardless of conditions (i.e., language and specific measures used), the Big Five domains would have similar correlations with the six domain scores of the ANPS measures.

The results of the present work are mostly in line with previous findings (Marengo et al., 2021). The associations between the long and short English-language variants of the ANPS and the Big Five personality traits show similar patterns as in the results/model presented by Montag and Panksepp (2017b) in terms of association magnitudes and directions.

With regards to SEEKING, the largest positive associations were observed with Openness to Experience and Extraversion, and the largest inverse associations were observed with Neuroticism. Of note, there are some ongoing discussions if SEEKING only robustly associates with Openness and/or Extraversion (Montag & Panksepp, 2017a). The results of the present study suggest that SEEKING can be associated both with Openness *and* Extraversion. Indeed, this is plausible in the light of Panksepp’s Affective Neuroscience Theory (Davis & Montag, 2019; Panksepp, 1998, 2011), because the neurobiology underlying the SEEKING system (e.g., the massive medial forebrain bundle, among others) is known to energize mammals/humans and to play a significant role in processing of rewards (and Extraversion is known to play a role in reward processing as well; Smillie, 2013).

Coherent with meta-analytic findings by Marengo et al. (2021), regarding the validation of the German ANPS-AR scales, high PLAY was moderately linked to higher Extraversion. Interestingly, the 4-item SEEKING scale was strongly correlated with higher Openness (*r* = .554; also mentioned above), and the 6-item SEEKING scale with a higher Cronbach’s alpha had a more moderate correlation (*r* = .461). Also, higher CARE was strongly associated with higher Agreeableness, although the 6-item CARE scale correlated only slightly higher than the 4-item scale (*r* = .558 to *r* = .545, respectively). As with SEEKING, CARE was also negatively correlated with Neuroticism. The negative ANPS-AR emotions were generally consistent with the meta-analysis by Marengo et al. (2021). ANGER, FEAR, and SADNESS were all linked to Neuroticism, with high ANGER also linking to low Agreeableness. SADNESS and Extraversion were strongly negatively correlated in both the English and German ANPS-AR versions (*r* = −.569 and *r* = −.526, respectively)^1^.

Given that primary emotional systems arise from subcortical areas of the mammalian brain (Davis & Montag, 2019), it has been proposed that these primary emotional systems function as “bottom-up drivers” of the lexically derived Big Five personality traits. In other words: from a neuroscientific perspective, one could hypothesize that these primary emotional systems influence the broader Big Five personality traits in a bottom-up way. However, whether that is the case is not in the scope of the current study, since our study does not encompass neuropsychological data and relies on self-reports and is cross-sectional in study design.

In datasets reported here, negative primary emotional systems (ANGER, FEAR, and SADNESS) have a strong positive association with Neuroticism, although ANGER has a smaller association than FEAR and SADNESS. This finding is also in line with results of the meta-analysis by Marengo et al. (2021). Considering the Affective Neuroscience Theory, these findings suggest that, in a higher order sense, Neuroticism might be “lumping” together all three of the ancient evolutionary emotional systems, even though each of these primary emotions can go along with unique clinical pathologies. The three positive primary emotions systems measured here are linked more uniquely to the Big Five, but there are still additional complexities that perhaps attest to the dynamic nature of how the primary emotions are expressed in real life.

Altogether, the results of the two present studies demonstrate the (external) validity of ANPS-AR. While Montag and Davis (2018) also presented validation data of the ANPS-AR with the ANPS in English language, it should be noted that some items as well as the response scale have been changed in more recent versions of the original ANPS-110. The results in Figure 1 (but also in correlation tables presented in Supplementary Materials) show that the similarities in association patterns were high and generally fit with the findings of the meta-analysis of Marengo et al. (2021).

In addition, the inclusion of four additional items to the German ANPS-AR improved the internal consistencies of subscales that demonstrated poor internal consistencies in the scale version without these variables. Based on this, the 28-item German ANPS-AR seems to be a tool that could provide similar results to its longer counterpart, potentially allowing for investigating the Affective Neuroscience Theory in a concise and efficient way. We hope that the current work inspires more researchers in the fields of psychology and neuroscience working on affective neuroscience to use this tool. To facilitate this, we have provided both the English and German-language ANPS-AR scales (alongside the instructions and rating scale) in the Supplementary Materials^2^.

Yet, the more unique use of the ANPS-AR lies in the realm of observer ratings of others where it is difficult for a third party to accurately rate contextual items or where contextual items may not be useful. Examples might be a therapist documenting a patient’s emotional responses or seeking to gain additional insight from the patient’s family members. However, even as adjectives may be the most natural way to describe behavior, that same transparency makes adjectives the easiest personality medium for “faking” responses in a desired direction such as clinical diagnostic assessments (which again may be countered by having ratings from additional observers) or applying for a job: all situations where the test taker has something significant to gain or lose (Cattell & Butcher, 1968). Still, in the end, adjectives used in appropriate circumstances may be capable of providing the most accurate objective assessment of personality.

## Footnotes

1 While item-level analyses were not in the main focus of the current study, we also explored the disparity in correlations between the 110-item and ANPS-AR SADNESS and BFI-44 Extraversion factors. These item-level correlations which are presented in Supplementary Materials show that, indeed, some particular items may be responsible for the observed associations.

2 It should be noted that the instructions used for the current study ANPS-AR scale had a slight difference from the scale instructions in the Supplementary Materials, as we improved the wording of the scales.

## Supplementary Materials

For supplementary material for this article, please visit https://doi.org/10.1017/pen.2021.6 click here to view supplementary material
