# Supplementary material for: The Affective Neuroscience Personality Scales: Linking the adjective and statement-based inventories with the Big Five Inventory in English and German-speaking samples
Source: Personal Neurosci. 2022 Mar 23;4:e7. doi: 10.1017/pen.2021.6 (PMC8988172; doi:10.1017/pen.2021.6)
Supplement: Supplementary file 1 [file S2513988621000067sup001.zip › S2513988621000067sup001.pdf]

Supplementary Materials A
ANPS-AR Questionnaires in ***German*** *language.*

Bitte beschreiben Sie, wie sehr die folgenden Worte auf Sie *generell* zutreffen.

		trifft überhaupt nicht zu	trifft nicht zu	trifft eher nicht zu	weder noch	trifft eher zu	trifft zu	trifft voll und ganz zu
zielstrebig	SEEKING +	1	2	3	4	5	6	7
ängstlich	FEAR +	1	2	3	4	5	6	7
fürsorglich	CARE +	1	2	3	4	5	6	7
hitzköpfig	ANGER +	1	2	3	4	5	6	7
lustig	PLAY +	1	2	3	4	5	6	7
oftmals traurig	SADNESS +	1	2	3	4	5	6	7
fantasielos	SEEKING -	7	6	5	4	3	2	1
nervös	FEAR +	1	2	3	4	5	6	7
gefühllos	CARE -	7	6	5	4	3	2	1
aggressiv	ANGER +	1	2	3	4	5	6	7
nicht verspielt	PLAY -	7	6	5	4	3	2	1
sozial unsicher	SADNESS +	1	2	3	4	5	6	7
dynamisch	SEEKING +	1	2	3	4	5	6	7
entspannt	FEAR -	7	6	5	4	3	2	1
(andere) pflegend	CARE +	1	2	3	4	5	6	7
nicht streitsüchtig	ANGER -	7	6	5	4	3	2	1
mache gerne Scherze	PLAY +	1	2	3	4	5	6	7
sozial selbstsicher	SADNESS -	7	6	5	4	3	2	1
neugierig	SEEKING +	1	2	3	4	5	6	7
grüblerisch	FEAR +	1	2	3	4	5	6	7
warmherzig	CARE +	1	2	3	4	5	6	7
temperamentvoll	ANGER +	1	2	3	4	5	6	7
humorvoll	PLAY +	1	2	3	4	5	6	7
sensibel gegenüber Zurückweisung	SADNESS +	1	2	3	4	5	6	7
umsorgend	CARE +	1	2	3	4	5	6	7
voller Energie	SEEKING +	1	2	3	4	5	6	7
wenig sympathisch	CARE -	7	6	5	4	3	2	1
motiviert	SEEKING +	1	2	3	4	5	6	7

Die grau markierten Items sind in der deutschsprachigen Version zur Verbesserung der Psychometrie hinzugekommen. Bei dem Item „(andere) pflegend“ wird darauf hingewiesen, dass in der Datenerhebung des Papers nur „pflegend“ verwendet wurde.

Auswertung: Die Punkte müssen für jede Skala aufaddiert werden.
Sollten Sie den Fragebogen als State-Maß einsetzen wollen, schlagen wir folgende Instruktion vor:
„Bitte beschreiben Sie, wie gut die folgenden Worte auf Sie *im Moment* zutreffen.“

Cite the German version of the ANPS-AR as follows: Rozgonjuk, D., Davis, K.L., Sindermann, C., and Montag, C. (2021) The Affective Neuroscience Personality Scales: linking the adjective and statement-based inventories with the Big Five Inventory in English- and German-speaking samples. *Personality Neuroscience*. doi: 10.1017/pen.2021.6

Cite the English version of the ANPS-AR as follows: Montag, C. & Davis, K. (2018) Affective Neuroscience Theory and Personality: An Update. *Personality Neuroscience*. *1*(e12), 1–12. doi: 10.1017/pen.2018.10

*ANPS-AR Questionnaire in **English** language.*

Please indicate how accurately the following words describe you *in general*.

		very inaccurate	inaccurate	slightly inaccurate	neither	slightly accurate	accurate	very accurate
purposeful	SEEKING +	1	2	3	4	5	6	7
anxious	FEAR +	1	2	3	4	5	6	7
caring	CARE +	1	2	3	4	5	6	7
hot-headed	ANGER +	1	2	3	4	5	6	7
funny	PLAY +	1	2	3	4	5	6	7
often sad	SADNESS +	1	2	3	4	5	6	7
unimaginative	SEEKING -	7	6	5	4	3	2	1
nervous	FEAR +	1	2	3	4	5	6	7
unsympathetic	CARE -	7	6	5	4	3	2	1
aggressive	ANGER +	1	2	3	4	5	6	7
not playful	PLAY -	7	6	5	4	3	2	1
socially insecure	SADNESS +	1	2	3	4	5	6	7
dynamic	SEEKING +	1	2	3	4	5	6	7
relaxed	FEAR -	7	6	5	4	3	2	1
nurturing	CARE +	1	2	3	4	5	6	7
not argumentative	ANGER -	7	6	5	4	3	2	1
jokes around	PLAY +	1	2	3	4	5	6	7
socially confident	SADNESS -	7	6	5	4	3	2	1
curious	SEEKING +	1	2	3	4	5	6	7
a worrier	FEAR +	1	2	3	4	5	6	7
warm	CARE +	1	2	3	4	5	6	7
temperamental	ANGER +	1	2	3	4	5	6	7
humorous	PLAY +	1	2	3	4	5	6	7
sensitive to rejection	SADNESS +	1	2	3	4	5	6	7

Scoring: The scores for each scale need to be summed up.
If you like to use the questionnaire as a state-measure, we propose the following instruction:
"Please indicate how accurately the following words describe you *at the moment*."

Cite the German version of the ANPS-AR as follows: Rozgonjuk, D., Davis, K.L., Sindermann, C., and Montag, C. (2021) The Affective Neuroscience Personality Scales: linking the adjective and statement-based inventories with the Big Five Inventory in English- and German-speaking samples. *Personality Neuroscience*. doi: 10.1017/pen.2021.6

Cite the English version of the ANPS-AR as follows: Montag, C. & Davis, K. (2018) Affective Neuroscience Theory and Personality: An Update. *Personality Neuroscience*. *1*(e12), 1–12. doi: 10.1017/pen.2018.10
