# Supplementary material for: The Affective Neuroscience Personality Scales: Linking the adjective and statement-based inventories with the Big Five Inventory in English and German-speaking samples
Source: Personal Neurosci. 2022 Mar 23;4:e7. doi: 10.1017/pen.2021.6 (PMC8988172; doi:10.1017/pen.2021.6)
Supplement: Supplementary file 1 [file S2513988621000067sup001.zip › S2513988621000067sup002.pdf]

Supplementary Materials

Supplementary Materials B

Correlation analysis results: Pearson correlations between the English-language BFI-44 and ANPS-AR variables (Study 1).

Variable	O	C	E	A	N	seek	fear	care	anger	play
**O**	1									
**C**	.136**	1								
**E**	.295***	.247***	1							
**A**	.074	.245***	.226***	1						
**N**	.002	-.325***	-.257***	-.320***	1					
**seeking**	.549***	.380***	.518***	.223***	-.249***	1				
**fear**	-.067	-.238***	-.297***	-.195***	.814***	-.258***	1			
**care**	.258***	.261***	.351***	.581***	-.151**	.387***	-.108*	1		
**anger**	.124**	-.146**	.222***	-.441***	.389***	.078	.266***	-.132**	1	
**play**	.258***	.096	.399***	.239***	-.203***	.356***	-.251***	.291***	.026	1
**sadness**	-.108*	-.320***	-.569***	-.276***	.667***	-.420***	.662***	-.282***	.097	-.281***

Notes. O = Openness to Experience; C = Conscientiousness; E = Extraversion; A = Agreeableness; N = Neuroticism. Lowercase ANPS variables are for ANPS-AR.

Supplementary Materials C

Correlation analysis results: Pearson correlations between the German-language BFI-44 and ANPS-AR variables (Study 2).

	**O**	**C**	**E**	**A**	**N**	**seek**	**seek28**	**SEEK**	**fear**	**FEAR**	**care**	**care28**	**CARE**	**anger**	**ANGER**	**play**	**PLAY**	**sad**
O	1																	
C	.074	1																
E	.245***	.123**	1															
A	.090*	.063	.115*	1														
N	-.029	-.221***	-.165***	-.157***	1													
seeking	.554***	.336***	.452***	.112*	-.182***	1												
seeking28	.461***	.415***	.528***	.144**	-.249***	.911***	1											
SEEKING	.492***	.276***	.410***	.111*	-.214***	.629***	.673***	1										
fear	.041	-.146**	-.250***	-.079	.765***	-.152**	-.224***	-.135**	1									
FEAR	-.010	-.172***	-.286***	-.039	.706***	-.200***	-.261***	-.187***	**.765******	1								
care	.187***	.065	.261***	.545***	.113*	.300***	.270***	.198***	.091*	.152**	1							
care28	.181***	.081	.280***	.558***	.086	.313***	.294***	.220***	.058	.137**	.971***	1						
CARE	.189***	-.005	.276***	.405***	.220***	.235***	.216***	.253***	.175***	.266***	.693***	.698***	1					
anger	.006	-.092*	.282***	-.401***	.367***	.084	.085	.050	.237***	.139**	-.077	-.106*	.054	1				
ANGER	-.030	-.127**	.142**	-.449***	.477***	-.018	-.031	-.083	.343***	.339***	-.088	-.105*	.048	.725***	1			
play	.323***	.034	.336***	.149**	-.076	.411***	.405***	.296***	-.055	-.081	.274***	.316***	.222***	.090*	.038	1		
PLAY	.164***	.083	.500***	.318***	-.265***	.339***	.417***	.396***	-.285***	-.253***	.347***	.394***	.346***	.019	-.081	.573***	1	
sadness	-.051	-.247**	-.526***	-.147**	.625***	-.313***	-.398***	-.300***	.670***	.679***	-.069	-.102*	.042	.121**	.258***	-.204***	-.445***	1
SADNESS	-.021	-.229**	-.167***	-.067	.664***	-.206***	-.266***	-.210***	.581***	.675***	.132**	.106*	.277***	.236***	.399***	-.075	-.248***	.647***

Notes. O = Openness to Experience; C = Conscientiousness; E = Extraversion; A = Agreeableness; N = Neuroticism. Lowercase ANPS variables are for ANPS-AR, while uppercase ANPS variables are for the 110-item ANPS. For SEEKING and CARE ANPS-AR, both the correlations with the 4 item and 6 item scales are presented.

Supplementary Materials D

Item-level Pearson correlations between SADNESS and Extraversion items (Study 2).

Variable	E1	E2	E3	E4	E5	E6	E7	E8
SADNESS1	-.032	-.156***	-.319***	-.042	-.106**	-.126***	-.259***	-.153**
SADNESS2	.030	-.033	-.072	.010	.023	-.131**	-.152***	-.042
SADNESS3	.014	-.102*	-.224***	-.052	-.022	-.077	-.239***	-.119**
SADNESS4	-.011	-.085	-.205***	-.053	.010	-.099*	-.160***	-.099*
SADNESS5	-.071	-.187***	-.249***	-.166***	-.114*	-.139**	-.274***	-.176***
SADNESS6	-.142**	-.046	-.151**	-.155***	-.089*	-.065	-.004	-.183***
SADNESS7	.022	-.021	.021	-.011	-.008	-.067	-.078	-.002
SADNESS8	.060	-.002	-.024	-.013	.044	-.159***	-.069	.055
SADNESS9	.026	-.047	-.168***	.003	-.006	-.033	-.205***	-.026
SADNESS10	.213***	.054	-.005	.076	.044	-.015	-.056	.127**
SADNESS11	.276***	.115*	.032	.112*	.138**	.041	.041	.251***
SADNESS12	.084	.010	-.001	.014	.067	-.064	-.074	.060
SADNESS13	.166***	.023	-.028	.088*	.093*	.014	-.103*	.124**
SADNESS14	-.444***	-.383***	-.261***	-.296***	-.395***	-.279***	-.291***	-.544***
sadness1	-.020	-.179***	-.293***	-.055	-.074	-.105*	-.279***	-.133**
sadness2	-.385***	-.493***	-.309***	-.293***	-.458***	-.375***	-.560***	-.524***
sadness3	-.396***	-.509***	-.304***	-.318***	-.442***	-.431***	-.545***	-.556***
sadness4	.006	-.104**	-.104**	.070	-.002	-.109*	-.252***	-.085

Notes. E = Extraversion. Lowercase sadness items are for ANPS-AR, while uppercase SADNESS items are for the 110-item ANPS. Correlation coefficients in grey font mark the associations that were non-significant.
